# Comparison of Antimicrobial Activity of Triple, Double, and Cefixime-Based Antibiotic Pastes Against Enterococcus Faecalis: An In Vitro Study

**DOI:** 10.7759/cureus.44024

**Published:** 2023-08-24

**Authors:** Jyoti Thakur, Sumit Mohan

**Affiliations:** 1 Pedodontics & Preventive Dentistry, Smile Sure Dental and Orthodontic Center, Ranchi, IND; 2 Conservative Dentistry and Endodontics, Dental College, Rajendra Institute of Medical Sciences (RIMS), Ranchi, IND

**Keywords:** colony forming units (cfu), antibacterial, calcium hydroxide, double antibiotic paste, triple antibiotic paste, cefixime, enterococcus faecalis (e. faecalis)

## Abstract

Aim: This *in vitro* study aims to compare the antimicrobial efficacy of triple antibiotic paste, double antibiotic paste, and cefixime-based triple antibiotic paste against *Enterococcus faecalis.*

Materials and methods: Fifty single-rooted, caries-free, permanent teeth without any developmental defects were included in this study. The specimens were divided into five groups, with each group consisting of 10 teeth that received a specific medicament. The groups were as follows: Group I: control; Group II: calcium hydroxide*; *Group III: triple antibiotic paste; Group IV: double antibiotic paste; and Group V: cefixime-based triple antibiotic paste. The antimicrobial activity of the medicaments was assessed against *E. faecalis* at the end of the seventh^ ^and 14^th ^days. The colony-forming units (CFU) were calculated using the Kolmogorov-Smirnov and Wilcoxon tests.

Results: After seven days of the experimental process, it was observed that the CFU count was highest in group I and lowest in group V. In a similar vein, after 14 days, the maximum decrease in CFU count was observed in Group V, while the least reduction in CFU count was observed in Group II. On intergroup comparison, it was found that the maximum decrease in CFU was noted in Group V, followed by Group IV, Group III, and Group II.

Conclusion: The study results indicated that the cefixime-enriched antibiotic paste had the greatest antimicrobial effectiveness, while the double and triple antibiotic pastes offered superior antibacterial efficacy against *E. faecalis *at the end of the seventh and 14^th^ days.

## Introduction

Microorganisms play an important role in dental diseases [1]. The root canal system comprises both aerobic and anaerobic microorganisms, and it is necessary to eliminate them as they are responsible for pulpal and periapical diseases [2]. The complexity of root canals makes it essential to rely on chemical and mechanical aids to ensure optimal sterilization of the infected root canal [3]. Biomechanical preparation, along with the use of irrigation and intracanal medicaments, can ensure a sterile root canal [3-5].

Over the years, several medicaments have been used in endodontic practice, of which calcium hydroxide has been considered the gold standard treatment [6]. However, it proves ineffective against *Enterococcus faecalis*, which is frequently encountered in retreatment cases. In such cases, the medicament of choice is triple antibiotic paste (TAP), which is composed of minocycline, ciprofloxacin, and metronidazole [1,7,8]. Nevertheless, the major drawback of minocycline is its tendency to cause coronal discoloration [9]. Double antibiotic paste (DAP) contains metronidazole and ciprofloxacin with the aim of reducing discoloration [2].

Cefixime is a third-generation broad-spectrum cephalosporin-based antibiotic that prevents the formation of bacterial cell walls by interfering with the production of peptidoglycans. This interference leads to a reduction in the stability of bacterial cell walls, ultimately causing bacterial cell lysis [10]. Cefixime-based antibiotic medication is an effective intracanal medicament.

Hence, the present in vitro study aims to compare the antibacterial efficacy of double, triple, and modified antibiotic pastes against *E. faecalis*.

## Materials and methods

Human mandibular premolars extracted from patients were used in the study. Radiographic evaluation was performed on all extracted premolars, and teeth with caries, root fissures, fractures, calcification, and multiple canals were excluded from the study. Teeth were scraped to remove debris, rinsed with distilled water, and stored in saline. The teeth were standardized to a length of 18 mm.

Access cavities were prepared using a high-speed bur (Dentsply Maillefer, Switzerland), and a size 10 K-file (Dentsply Maillefer, Switzerland) was placed to ensure canal patency. Samples were prepared 1 mm short of the apex using Protaper F3 Files (Dentsply Maillefer, Switzerland). The root canals were disinfected using 5.25% sodium hypochlorite and 17% EDTA. The sealed root apices were sterilized at 121^0^C for 20 minutes at 20 psi pressure in an autoclave. A 15 µL bacterial suspension of *E. faecalis* was inoculated into each canal. For optimal penetration of bacteria into the dentinal tubules, the samples were incubated at 36.5°C for six hours.

The experimental antibiotic paste used in this study consisted of a combination of minocycline, ciprofloxacin, metronidazole, and cefixime tablets. The paste was prepared according to the methodology described by Hoshino et al. [11]. The enteric coating of minocycline, ciprofloxacin, metronidazole, and cefixime tablets was removed using a slow-speed handpiece with a carbide bur. The medications were then pulverized using a porcelain mortar and pestle to achieve a fine powder. The experiment involved weighing 100 mg of each medicament using a high-precision electronic balance and mixing in equal ratios with 10 ml of sterile water to obtain a TAP, DAP, and cefixime-enriched antibiotic paste. Calcium hydroxide was also tested against *E. faecalis*. The specimens were divided into five groups (Table 1).

**Table 1:** Groups made in the study

GROUP	n	Medicament
I	10	Negative Control
II	10	Calcium hydroxide
III	10	Triple Antibiotic Paste
1V	10	Double Antibiotic Paste
V	10	Cefixime enriched Triple Antibiotic Paste

All the samples were introduced into a canal, isolated, and incubated for seven days. After seven days, a hole was prepared using a #04 round carbide bur (Teeskavan Co., Tehran, Iran) on each root, 3 mm short of the apex. The dentinal shavings obtained were allowed to fall into Brain Heart Infusion (BHI) broth, which was then incubated at 36.5°C for 24 hours. A colony count of *E. faecalis* was performed, and the above procedure was repeated after 14 days. The Wilcoxon test (a non-parametric statistical test) was used to determine intergroup variation.

## Results

The mean values of colony-forming units (CFU) varied significantly (P<0.001) among the five groups. CFU was greater in Group I (control) than in the study groups. The mean CFU count of Group I at the end of the seventh day was the highest, followed by Group II (calcium hydroxide), and the least CFU count was observed in Group V (cefixime-modified TAP) (Table 2).

**Table 2:** Colony forming units (CFU) on the seventh day

Group	Medicament	Range	Median	Mean	SD
I	Control	1700 to 3300	2350.00	2433.33	571.02
II	Calcium hydroxide	800 to 1700	1200.00	1250.00	284.45
III	Triple Antibiotic Paste	400 to 1200	825.00	791.67	263.57
IV	Double Antibiotic Paste	1 to 300	38.50	97.33	119.91
V	Cefiximemodified Triple Antibiotic Paste	1 to 25	2.50	6.92	7.75

By conducting multiple comparisons, it was observed that the CFU were higher in Group I (control) compared to Group IV (DAP) and Group V (cefixime-modified TAP). Moreover, the maximum decrease in CFU count was observed in Group V, while the least reduction in CFU count was observed in Group II (Table 3). 

**Table 3:** Colony forming units (CFU) on the 14th day

Group	Medicament	Range	Median	Mean	SD
I	Control	2900 to 5200	4000.00	4016.67	792.96
II	Calcium hydroxide	300 to 1700	1100.00	1108.33	450.17
III	Triple Antibiotic Paste	200 to 1200	550.00	625.00	330.63
IV	Double Antibiotic Paste	0 to 55	0.00	8.58	16.42
V	Cefiximemodified Triple Antibiotic Paste	0 to 7	0.00	0.58	2.02

Intergroup variation was calculated using the Wilcoxon test for all groups at seven and 14 days, respectively, in Table 4. A maximum decrease in CFU was observed in Group V (cefixime-modified triple antibiotic paste), followed by Group IV (double antibiotic paste), Group III (triple antibiotic paste), and Group II (calcium hydroxide) (Table 4).

**Table 4:** Inter group comparison of medicaments

Time	Group I	Group II	Group III	Group IV	Group V
7^th^ day	2433.33 ± 571.02	1250.00 ± 284.45	791.67 ± 263.57	97.33 ± 119.91	6.92 ± 7.75
14^th^ day	4000.00 ± 4016.67	1100.00 ± 1108.33	550.00 ± 625.00	8.58 ± 16.425	0.58 ± 2.02

## Discussion

An infected root canal system harbors various microorganisms, especially in teeth with persistent disease. *Enterococci* have a high prevalence, followed by *Streptococci*, *Lactobacilli*, and *Actinomyces* species [12-14]. *E. faecalis* is a resilient bacterium found in root canals that exhibits both intrinsic and acquired resistance to multiple antibiotics. It also shows reduced sensitivity to penicillin and other beta-lactam drugs [15]. Cefixime, being a cephalosporin antibiotic with a penicillin-like foundation, calls for a judicious examination of its potential interactions and implications within the context of root canal disinfection. Cefixime's interactions with the other antibiotics in TAP, as well as their combined effects on clinical outcomes, call for a complex analysis that goes beyond the existing parameters of this investigation. By inhibiting beta-lactamase, cefixime can enhance the susceptibility of *E. faecalis* to the other antibiotic components within TAP. This synergistic effect may lead to improved treatment outcomes, especially against bacteria that exhibit resistance to conventional therapies. Therefore, it is important to develop strategies to control infections caused by this organism. Hence, *E. faecalis* was chosen for the present study.

Intracanal medicaments play a major role in maintaining a sterile root canal between appointments. Although a plethora of intracanal medicaments exist, each has its advantages and disadvantages [16]. Currently, calcium hydroxide is used to treat the infection caused by this organism. Therefore, calcium hydroxide was also used as a test medication. In their study, Jenks et al. [17] confirmed that TAP or DAP provides superior root canal disinfection compared to calcium hydroxide, which is consistent with the findings of the present study.

Triple, double, or modified antibiotic intracanal medicaments also have a few disadvantages. Metronidazole has been known to possess allergic potential, which may be more likely if the medication is extruded from the apical foramen during administration [18]. Few studies have compared the effectiveness of ciprofloxacin, metronidazole, and cefixime. In comparison, the detrimental effects of cefixime are generally mild and short-term, with rare hypersensitivity reactions [19]. Hence, it can be safely used in pediatric patients. In the absence of inter-appointment medicaments in multi-visit endodontics, bacteria tend to survive and thrive during instrumentation and irrigation [20,21]. Hence, the importance of intracanal medicament cannot be underestimated in multiple-visit endodontics. In the present study, even after 14 days, the cefixime-modified TAP demonstrated superior antibacterial efficacy compared to calcium hydroxide. Works by Hoshino et al. [11] and Sato et al. [22] suggest that desired antibacterial efficacy is possible even at low concentrations of antibiotics. Conflicting results from other studies may be attributed to various factors, including differences in study designs, variations in disinfection protocols, the use of different irrigating solutions and regimens, variations in the application time of medicaments, and differences in the transport media used.

The study limitations include a small sample size; and few medicaments checked. Also, it is an in vitro study, so a clinical check of the efficacy has to be done in future studies.

## Conclusions

Within the limitations of the study, it was observed that while calcium hydroxide is considered the gold standard treatment for root canal disinfection, it is not as effective in retreatment cases where *E. faecalis* is predominant. In light of this, the integration of cefixime-enriched triple antibiotic paste (TAP) emerges as a prudent consideration for clinical implementation. This proposition is founded upon several factors: the heightened antibacterial efficacy demonstrated against *E. faecalis*, the propensity for mild irritation, and the negligible occurrence of discoloration.

## References

[1] Menezes MM, Valera MC, Jorge AO, Koga-Ito CY, Camargo CH, Mancini MN (2004). In vitro evaluation of the effectiveness of irrigants and intracanal medicaments on microorganisms within root canals. Int Endod J.

[2] Vijayaraghavan R, Mathian VM, Sundaram AM, Karunakaran R, Vinodh S (2012). Triple antibiotic paste in root canal therapy. J Pharm Bioallied Sci.

[3] Yaduka P, Sharma S (2014). Novel intracanal medicaments and its future scope. Int J Pharm Biol Sci.

[4] Yoldaş O, Doğan C, Seydaoğlu G (2004). The effect of two different calcium hydroxide combinations on root dentine microhardness. Int Endod J.

[5] Bansal R, Jain A (2014). Overview on the current antibiotic containing agents used in endodontics. N Am J Med Sci.

[6] Athanassiadis B, Abbott PV, Walsh LJ (2007). The use of calcium hydroxide, antibiotics and biocides as antimicrobial medicaments in endodontics. Aust Dent J.

[7] Abdel Hamid E, Abdel Aziz S, Sadek HS, Ibrahim AM (2018). Effectiveness of triple antibiotic paste as an intra-canal medication for the root canal treatment of non-vital teeth with apical periodontitis: a systematic review. F1000Res.

[8] Zargar N, Rayat Hosein Abadi M, Sabeti M, Yadegari Z, Akbarzadeh Baghban A, Dianat O (2019). Antimicrobial efficacy of clindamycin and triple antibiotic paste as root canal medicaments on tubular infection: An in vitro study. Aust Endod J.

[9] Kim JH, Kim Y, Shin SJ, Park JW, Jung IY (2010). Tooth discoloration of immature permanent incisor associated with triple antibiotic therapy: a case report. J Endod.

[10] Rödig T, Hirschleb M, Zapf A, Hülsmann M (2011). Comparison of ultrasonic irrigation and RinsEndo for the removal of calcium hydroxide and Ledermix paste from root canals. Int Endod J.

[11] Hoshino E, Kurihara-Ando N, Sato I, Uematsu H, Sato M, Kota K, Iwaku M (1996). In-vitro antibacterial susceptibility of bacteria taken from infected root dentine to a mixture of ciprofloxacin, metronidazole and minocycline. Int Endod J.

[12] Sundqvist G, Figdor D (2003). Life as an endodontic pathogen. Etiological differences between untreated and filled root canals. Endod Top.

[13] Peters LB, Wesselink PR, Buijs JF, van Winkelhoff AJ (2001). Viable bacteria in root dentinal tubules of teeth with apical periodontitis. J Endod.

[14] Peciuliene V, Maneliene R, Balcikonte E, Drakteinis S, Rutkunas V (2008). Microorganisms in root canal infections: a review. Stomatol Baltic Dent Maxillofac.

[15] Heath CH, Blackmore TK, Gordon DL (1996). Emerging resistance in Enterococcus spp. Med J Aust..

[16] Pavaskar R, Chalakkal P, Krishnan R, Sirikonda S, Vasepalli M, Venkataramana P (2014). Study comparing the effectiveness of chlorhexidine, calcium hydroxide and linezolid based medicaments against enterococcus faecalis. J Clin Diagn Res.

[17] Jenks DB, Ehrlich Y, Spolnik K, Gregory RL, Yassen GH (2016). Residual antibiofilm effects of various concentrations of double antibiotic paste used during regenerative endodontics after different application times. Arch Oral Biol.

[18] Kaufman AY, Solomonov M, Galieva D, Abbott PV (2014). Allergic reaction to the tetracycline component of Ledermix paste: a case report. Int Endod J.

[19] Tan BJ (1995). Cefixime use in children: when and why. Can J Infect Dis.

[20] Byström A, Sundqvist G (1981). Bacteriologic evaluation of the efficacy of mechanical root canal instrumentation in endodontic therapy. Scand J Dent Res.

[21] Bystrom A, Sundqvist G (1985). The antibacterial action of sodium hypochlorite and EDTA in 60 cases of endodontic therapy. Int Endod J.

[22] Sato I, Ando-Kurihara N, Kota K, Iwaku M, Hoshino E (1996). Sterilization of infected root-canal dentine by topical application of a mixture of ciprofloxacin, metronidazole and minocycline in situ. Int Endod J.

